# Supplementary material for: Co-Isolation and Characterization of Two Pandoraviruses and a Mimivirus from a Riverbank in Japan
Source: Viruses. 2019 Dec 4;11(12):1123. doi: 10.3390/v11121123 (PMC6950457; doi:10.3390/v11121123)
Supplement: Supplementary file 1 [file viruses-11-01123-s001.zip › Supplementary_Materials.pdf]

Supplementary Materials for

Co-isolation and characterization of two pandoraviruses and a mimivirus from a riverbank in Japan

Motohiro Akashi [1,*] and Masaharu Takemura [1,*]

[1] Laboratory of Biology, Department of Liberal Arts, Faculty of Science, Tokyo University of Science, Shinjuku, Tokyo 162-8601, Japan

* Correspondence: motoa@rs.tus.ac.jp (M.A.), takemura@rs.kagu.tus.ac.jp (M.T.)

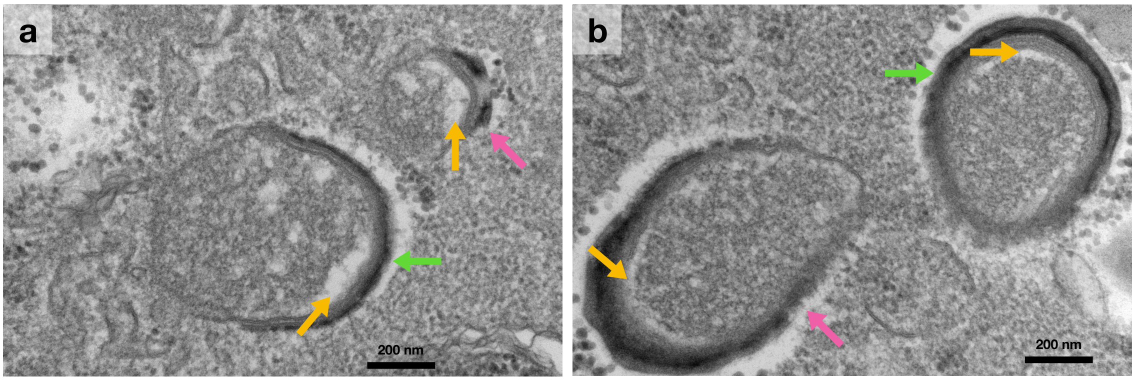

FIG S1 Pandoravirus morphogenesis started at the end of the viral particle that is opposite the ostiole-like apex. (**a**) *Pandoravirus hades*; (**b**) *P. persephone*. Scales are shown in all figures. Pink arrows: morphogenesis started at the ostiole-like apex. Green arrows: morphogenesis started at the opposite end of the ostiole-like apex. Yellow arrow: vacant space between the partially formed capsid and nucleocapsid.

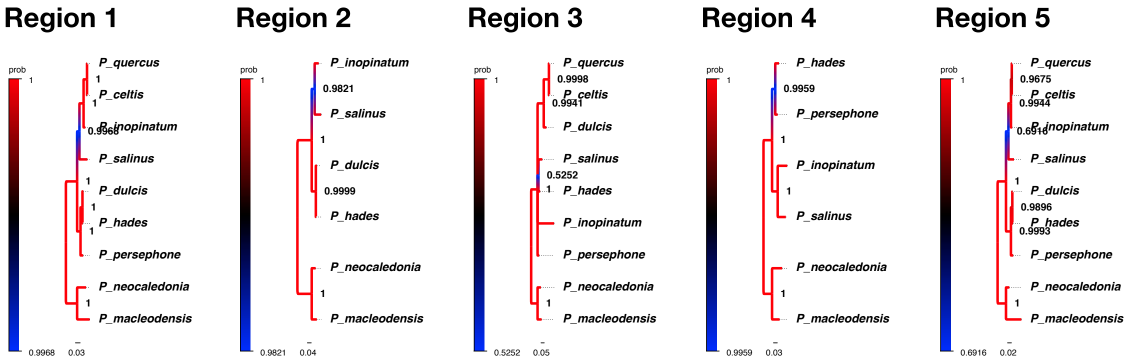

FIG S2 Bayesian phylogenetic trees of the specific regions of *polB*. Regions correspond to the *polB* alignment regions shown in Fig. 3. Color scale and node number: posterior probability. Scale bar: number of substitutions per site.

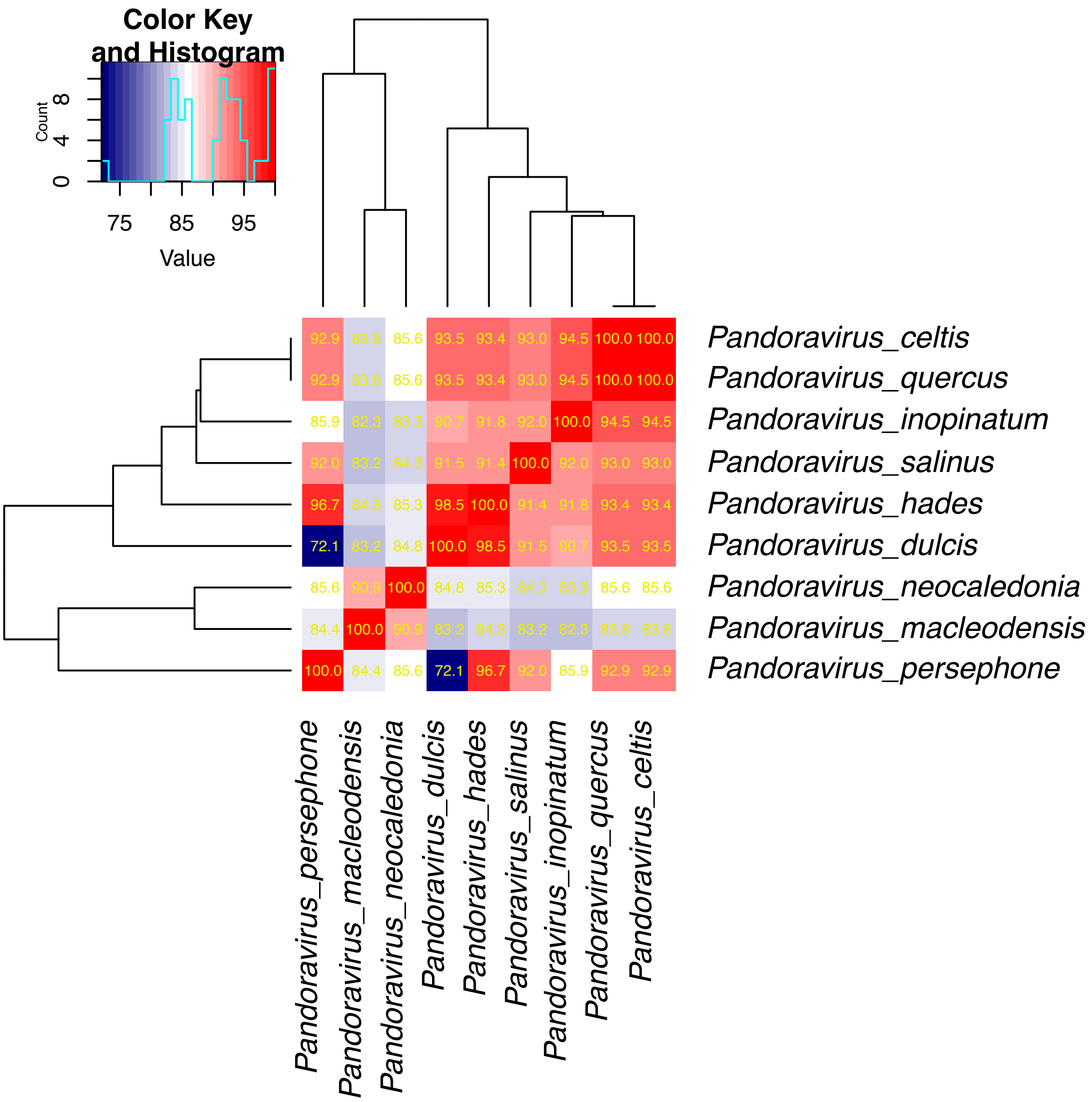

FIG S3 Heatmap of pairwise identities (%) among the *polB* sequences of pandoraviruses. Heatmap colors correspond to those shown in the key at the top left, in which a histogram of values is also shown. The pairwise identity values are given in yellow in each square.

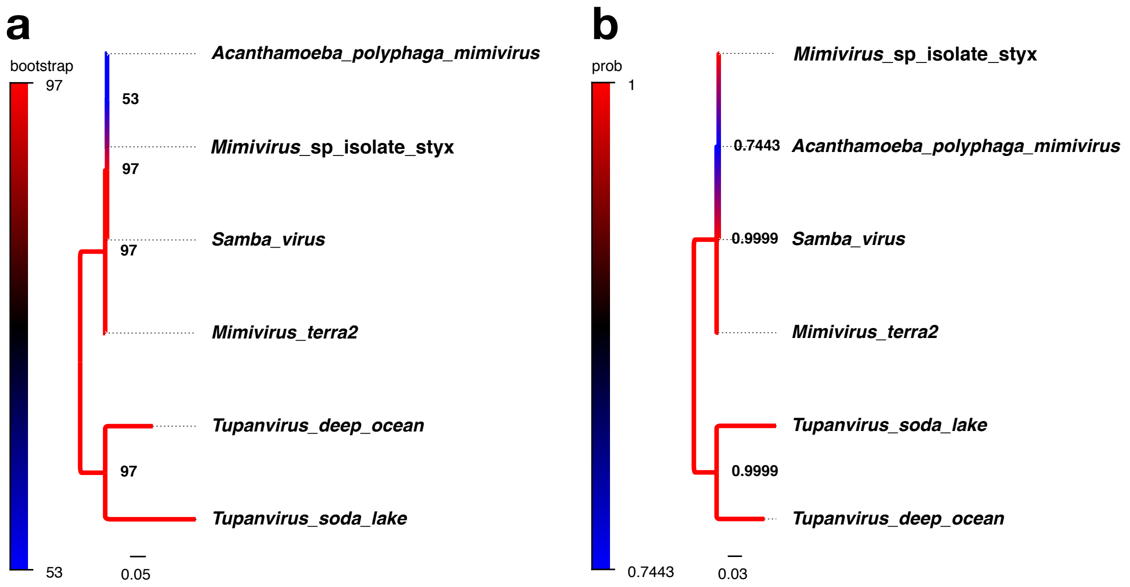

FIG S4 Phylogenetic analysis of the *polB* gene of members of the *Mimiviridae* family. (**a**) Maximum likelihood phylogenetic tree. Color scale and node number: bootstrap value. (**b**) Bayesian phylogenetic tree. Color scale and node number: posterior probability. Scale bar: number of substitutions per site.

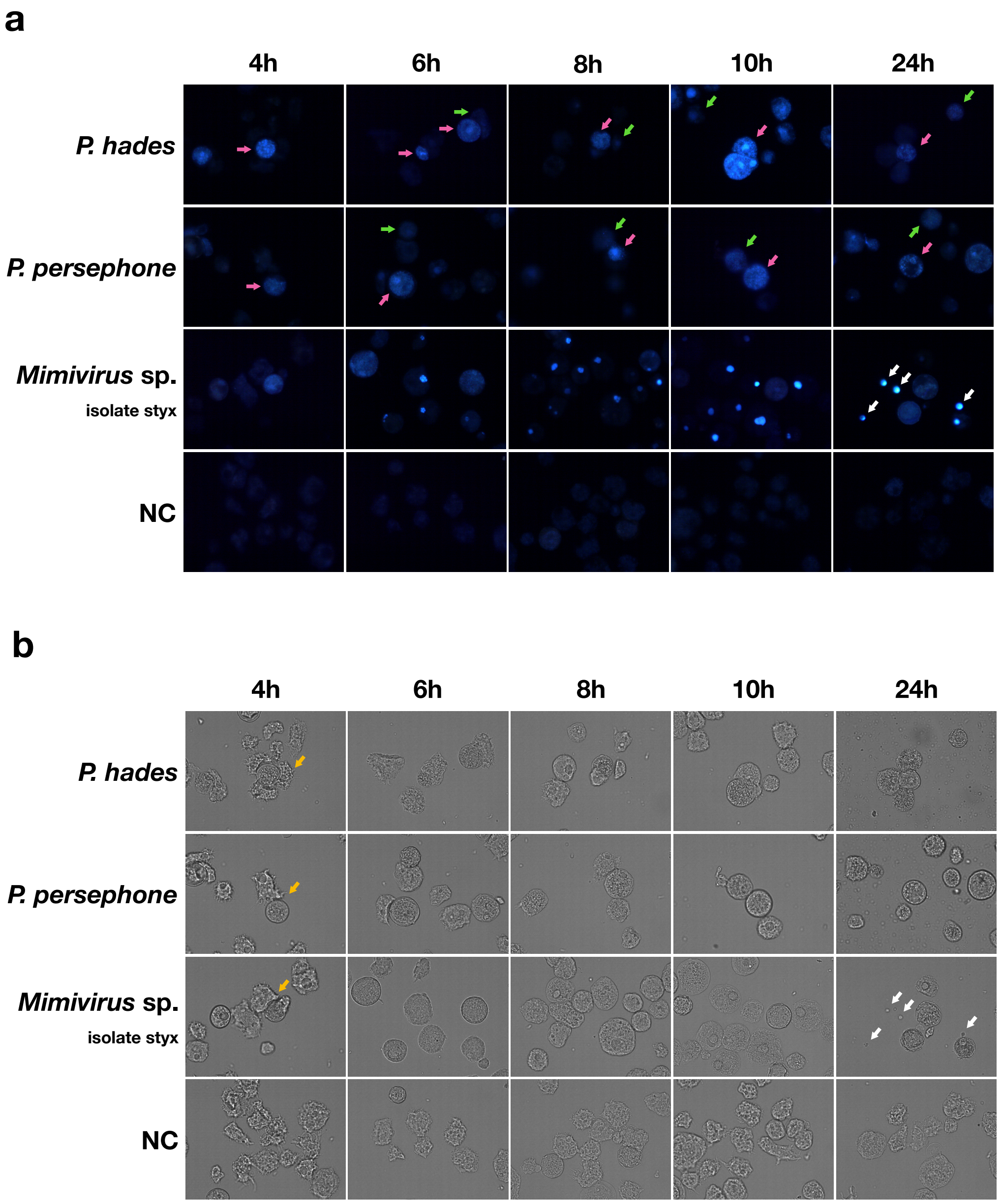

FIG S5 DAPI staining analysis of *P. hades*, *P. persephone*, and *Mimivirus* sp. isolate styx (Unmerged). (**a**) DAPI (4'6-diamidino-2-phenylindole). (**b**) Light. Green arrows: examples of partially stained cells. Pink arrows: examples of well-stained cells. White arrows: free virus factories. Yellow arrows: amoeba adhering to cells that exhibit cytopathic effects. NC: Uninfected amoeba cells as a negative control. Objective lens: x40.

Typing/sequencing analysis		Primer name	forward/reverse	sequence (5'-3')
	Pandoravirus	P1	for	GTGAGCGTCAAGGGCATG
		P2*	for	CCAAACCTCGCACCACTTTG
		P3	for	CGAGGGTCTATGTGATGCAGTG
		P4	for	ACCCATCCACAGAGAACTGG
		P5	for	CTACGATGCCATGTTTGCGG
		P6	for	CGATCGAGGTGTGGACCGAG
		P7	for	GCAACGACCAGACCTACGTGC
		P8	rev	CATCCACTGGGTGATCGGCGCC
		P8	rev	GGCGCCGATCACCCAGTGGATG
		P9	rev	CATCCACTGGGTGATCGGCGCC
		P10	rev	TCGAGGAGGCCACTCACATA
		P11	rev	GTGCATAAATGCGGTGATTG
		P12*	rev	TCGAGCGTGTACGATTCGAG
		P13	rev	CAAAGGCGTCCGTGTTGTAGC
	P. hades	Phad_1	for	CCTGCCGCTCATGCTCATCAG
		Phad_2	for	GTCTTTGTGCAGCACGTGCAG
		Phad_3	rev	AGGTTGCGTCAGACCATGATACATG
		Phad_4	rev	GTAAGTTTGTTCCTTCTGGCGATGTAGTG
	P. persephone	Ppers_1	for	GAGGAGATCTTTGAGCACTATGCGTC
		Ppers_2	rev	ACCCTCTTGCGCTGGTTCTTGAG
	Mimivirus	M1*	for	GAGACGGATCATGTGGTTCCT
		M2*	rev	GAAGTGTCAAAGGGCTGCCA

RAPD analysis		Primer name		sequence (5'-3')
		RAPD_1		CAATCGCCGT
		RAPD_2		AGGGGTCTTG
		RAPD_3		GAAACGGGTG
		RAPD_4		AGGTGACCGT
		RAPD_5		CAAACGTCGG
		RAPD_6		GTTGCGATCC

TABLE S1 PCR primers used in this study. Forward (for)/reverse (rev): primer directions based on *polB* gene coding direction. Primer names with asterisk: primers used for initial screening.

VIDEO S1 Lysis of *Acanthamoeba castellanii* cells infected with *Mimivirus shirakomae* and virus factory (VF) evacuation. (8 sec~): The small sphere (probable VF) appears immediately after lysis of the *A. castellanii* cell that exhibits cytopathic effects.
